# Cerebral Autosomal Dominant Arteriopathy With Subcortical Infarcts and Leukoencephalopathy (CADASIL): A Rare Cause of Transient Ischemic Attack

**DOI:** 10.7759/cureus.30940

**Published:** 2022-10-31

**Authors:** Shoaib Ashraf, Nishant Allena, Elina Shrestha, Manjeet Dhallu, Misbahuddin Khaja

**Affiliations:** 1 Internal Medicine, BronxCare Health System, Bronx, USA; 2 Neurology, BronxCare Health System, Bronx, USA

**Keywords:** stroke in young patients, leukoencephalopathy, notch 3 gene, migraine, stroke, cerebral autosomal dominant arteriopathy with subcortical infarcts and leukoencephalopathy (cadasil)

## Abstract

Cerebral autosomal dominant arteriopathy with subcortical infarcts and leukoencephalopathy (CADASIL) is increasingly recognized as an inherited and autosomal dominant arteriopathy of the cerebral vasculature, which is commonly misdiagnosed due to its different modes of presentation. It is characterized by variable manifestations of ischemic episodes, migraine with aura, cognitive deficits, and psychiatric disturbances. CADASIL is caused by a genetic mutation in the *NOTCH3* gene, which is present on chromosome 19.

The diagnosis of CADASIL can be made by personal and family history, skin biopsy, and magnetic resonance imaging (MRI) of the head showing high-intensity signal lesions, microbleeds, and white matter changes. There are currently no disease-modifying therapies available for CADASIL, and management focuses on reducing risk factors such as diabetes and hypertension and control of symptoms. We present a rare cause of transient ischemic attack (TIA) in a young female who was later diagnosed with CADASIL and aim to highlight rare and inherited causes of TIA and strokes in younger patients.

## Introduction

Cerebral autosomal dominant arteriopathy with subcortical infarcts and leukoencephalopathy (CADASIL) is a hereditary autosomal dominant arteriopathy of the brain, which is commonly misdiagnosed due to its different modes of presentation. It is characterized by variable manifestations of recurrent stroke or transient ischemic attack (TIA), migraine with aura, and vascular dementia at a younger age. *NOTCH3* is the culprit gene in CADASIL, but genetic testing for it is a time-consuming process. The mutations of the *NOTCH3* gene can be at multiple sites on chromosome 19 [1], and it encodes a large transmembrane receptor, which is expressed in arterial smooth muscle cells. The diagnosis of CADASIL can be made by personal and family history, skin biopsy showing the accumulation of granular osmiophilic material (GOM) in the media of arterioles, and magnetic resonance imaging (MRI) of the head showing high-intensity signal lesions, microbleeds, and white matter changes. Here, we present a rare cause of transient ischemic attack in a young female who was later diagnosed with CADASIL.

## Case presentation

A 33-year-old female with medical comorbidities of migraine came to the hospital with complaints of right upper and lower extremity paresthesia. The patient was driving when she first noticed numbness of the right thumb, which later progressed to the right upper and lower extremities, the right side of the face, and a brief episode of aphasia.

In the emergency department, she was anxious, alert, and oriented to time, place, and person. Her vital signs upon arrival were heart rate of 78 beats/minute, blood pressure of 128/82 mmHg, respiratory rate of 18 breaths/minute, temperature of 98.1°F, and oxygen saturation of 99% in room air. Her detailed neurological examination included intact extraocular eye movement with pupils equal, round, and reactive to light and accommodation on both eyes, with no nystagmus. Her tongue was midline, her speech was fluent, her gait was normal with no ataxia noted, no drift was present on upper and lower extremities on both sides, motor examination revealed 5/5 strength on all muscle groups on both sides, and decreased sensation was noted to light touch and pinprick on right side of the face, right upper extremity, and right lower extremity. Her National Institutes of Health (NIH) Stroke Scale was 1, for right-sided paresthesia.

**Figure 1:**
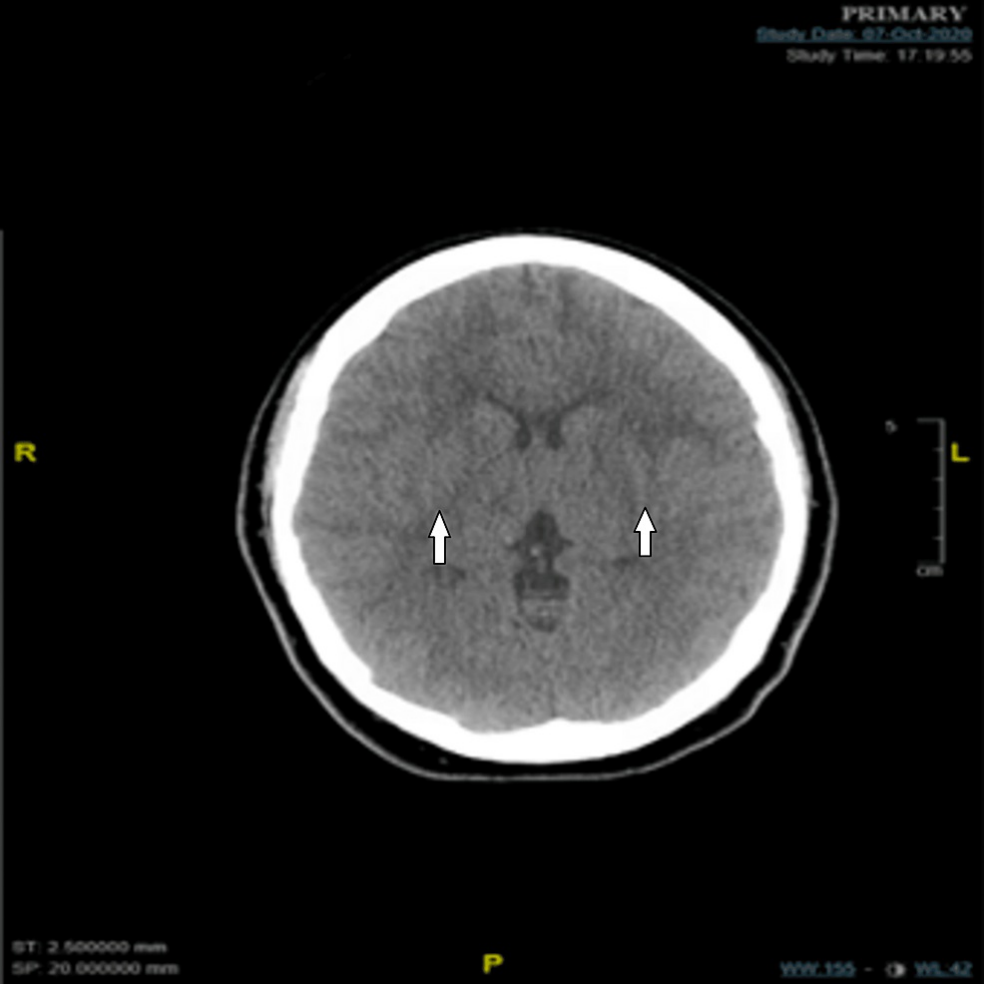
Computerized tomography (CT) of the head without contrast White arrows showing mild bilateral white matter hypoattenuation

Stroke code protocol was activated, and the patient was taken for computerized tomography (CT) scan of the head. CT of the head without contrast showed no evidence of acute territorial infarcts or hemorrhage, but there were mild bilateral white matter hypoattenuation and temporal lobe encephalomalacia (Figure 1).

Tissue plasminogen activator (tPA) was deferred due to mild and improving symptoms and the presence of white matter hypoattenuation on the CT scan. Her basic laboratory findings were within the normal limit except for anemia (Table 1). Chest X-ray was normal, an electrocardiogram showed normal sinus rhythm with a heart rate of 71 beats/minute, and urine toxicology was negative. The laboratory test values done on the day of presentation are listed in Table 1.

**Table 1:** Basic laboratory tests

Laboratory parameter	Admission day	Reference range
White blood cell count (per μl)	7.5	4.8-10.8
Hemoglobin (g/dl)	10.8	11-16
Platelet (per μl)	451	150-400
Sodium (mEq/L)	140	135-145
Glucose (mg/dL)	105	120-170
Prothrombin time/international normalized ratio (seconds)	11.6/0.98	9.9-13.3/0.90-1.09
Activated partial thromboplastin time (seconds)	29.4	27.2-39.6

**Figure 2:**
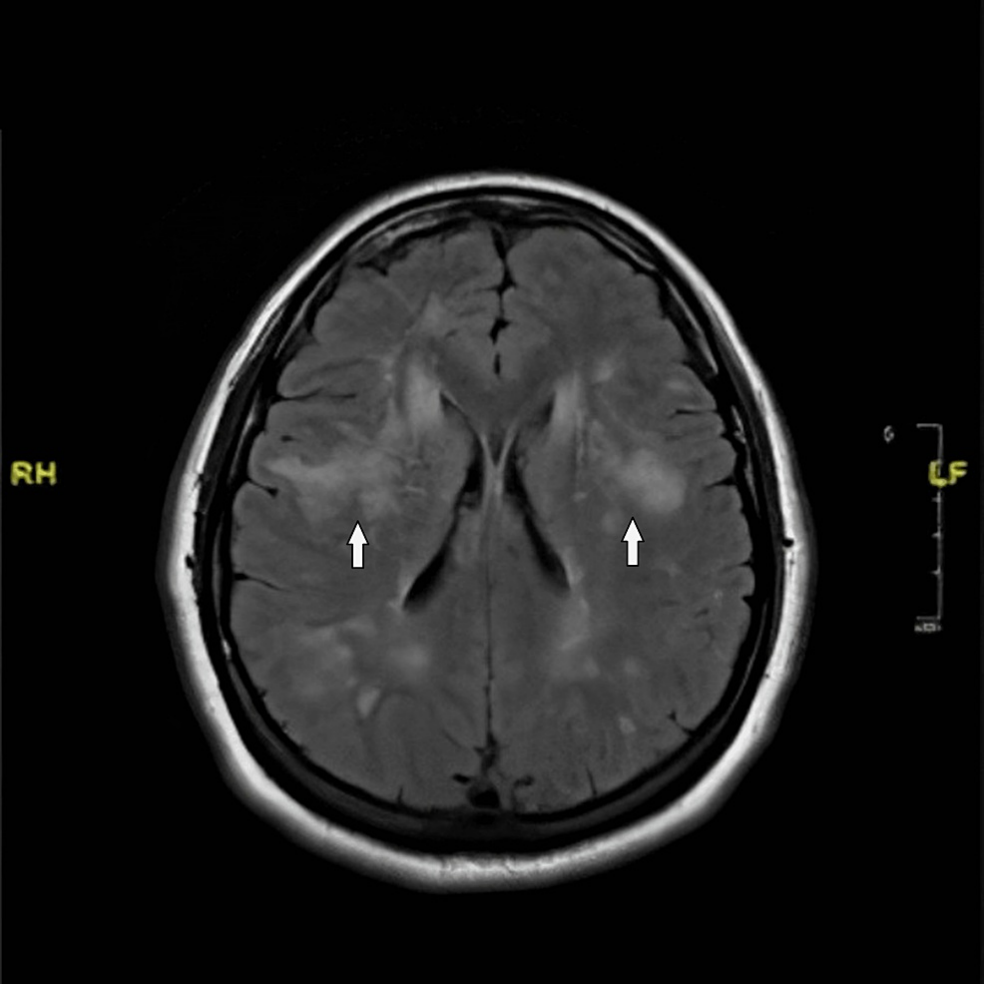
MRI of the brain (T2/FLAIR) White arrows show diffuse, disseminated, confluent, relatively symmetric white matter signal disease process involving periventricular and prominently subcortical white matter tracts MRI: magnetic resonance imaging; FLAIR: fluid-attenuated inversion recovery

She remained asymptomatic in the emergency department and was transferred to the floor in six hours. Specific anticoagulation tests, including protein C test, protein S test, prothrombin gene analysis, antithrombin III assay, factor V Leiden mutation analysis, cardiolipin gene antibody screen, and lupus anticoagulant evaluation, were all negative. Her echocardiogram revealed an ejection fraction of 72%, normal left ventricle structure and function, and normal valve structure and function. Doppler of the carotid arteries did not show any hemodynamically significant stenosis in bilateral internal carotid arteries. However, magnetic resonance imaging (MRI) of the head revealed acute disseminated encephalomyelitis confluent relatively symmetric white matter signal disease process involving periventricular and prominently subcortical white matter tracts but no associated abnormal diffusion signal or abnormal postcontrast enhancement (Figure 2).

An extensive review of her family history revealed that her mother had multiple strokes and was diagnosed with CADASIL syndrome. The test for mutations in the *NOTCH3* gene was sent, which identified a heterozygous missense pathologic variant, c.505C>T (p.Arg169Cys). CADASIL diagnosis was made by the cumulative clinical presentation, imaging studies, and, most importantly, genetic testing. The patient was discharged on aspirin and atorvastatin. She followed up with neurology as an outpatient, where her prognosis was explained, and medical management was continued.

## Discussion

Cerebral autosomal dominant arteriopathy with subcortical infarcts and leukoencephalopathy (CADASIL) is an autosomal dominant inherited cerebrovascular disorder that is an important cause of stroke in young patients caused by mutations in the *NOTCH3* gene on chromosome 19 [1]. As mentioned above, with the inheritance being autosomal dominant, each child has a 50% (one in two) chance of inheriting a mutated copy of the gene. Studies have shown that CADASIL is believed to have 100% penetrance [2]. Information on the prevalence of CADASIL has mostly originated from European registries, which put the prevalence between two and four per 100000 [3]. However, recent studies have found an increased frequency of *NOTCH3* cysteine-altering pathogenic variants at one in 300 in the general population with the highest frequency of one in 100 people of Asian descent [4]. These studies have shown that the prevalence of CADASIL is much more prevalent than what was initially estimated, approximately 100-fold higher with a much broader spectrum ranging from classic CADASIL to milder small vessel disease.

The syndrome is mainly caused by cysteine-altering pathologic variants in the *NOTCH3* gene, leading to vasculopathy changes involving the small penetrating arteries, arterioles, and brain capillaries. To establish a diagnosis of CADASIL, either documentation of a typical *NOTCH3* pathogenic variant by genetic analysis or documentation of characteristic ultrastructural deposits within small blood vessels by skin biopsy is required. Gene testing can be done by *NOTCH3* sequence analysis or gene-targeted deletion. However, because genetic screening does not detect all patients with CADASIL, a skin biopsy is recommended in cases where there is a high index of clinical suspicion for CADASIL. Granular osmiophilic material (GOM) [5] within the vascular basal lamina of arteries, arterioles, and precapillaries on electron microscopy and deposition of the extracellular domain of the *NOTCH3* receptor in the vascular media of arteries and arterioles are the characteristic changes seen. The *NOTCH3* gene testing done on our patient revealed a heterozygous missense pathologic variant, c.505C>T (p.Arg169Cys). *NOTCH3* sequencing test is highly reliable with a >95% sensitivity and 100% specificity [6].

CADASIL syndrome exhibits a broad spectrum of clinical symptoms, which include migraines, ischemic strokes and transient ischemic attack (TIA), cognitive defects, neuropsychiatric defects, and reversible encephalopathy. Recurrent ischemic episodes (TIA or stroke) were the most frequent presentation found in 71% of the cases (mean age at onset: 46.1 years; range: 30-66 years; SD: 9.0 years) [7]. Forty-eight percent of the cases had developed cognitive deficits. Dementia (28%) was frequently accompanied by gait disturbance (90%), urinary incontinence (86%), and pseudobulbar palsy (52%). Thirty-nine patients (38%) had a history of migraine (mean age at onset: 26.0 years; SD: 8.2 years), which was classified as migraine with aura in 87% of the cases [7]. Psychiatric disturbances were present in 30% of the cases, with adjustment disorder (24%) being the most frequent diagnosis. Ten percent of patients had a history of epileptic seizures [7]. Our patient suffered from occasional migraine attacks without aura and came in with right upper limb paresis that resolved in under two hours and a brief episode of aphasia, which lasted for a few minutes.

Obtaining a thorough family history, especially in patients presenting with a stroke/TIAs at a young age, is an important tool to diagnose the cause of the presenting complaints in this population. It played an important role in diagnosing our patient as she gave a history of her mother having multiple strokes at a very early age. MRI findings typically include anterior temporal lobe white matter hyperintensities visualized on T2-weighted sequences and are found in 90% of patients with CADASIL; external capsule and corpus callosum hyperintensities on T2-weighted sequences are also a characteristic of this condition [8]. Cerebral microhemorrhages are seen on T2-weighted images in 31%-69% of the patient population [9,10]. Atrophic changes of the brain parenchyma are another important MRI finding [11]. An MRI of the brain obtained on our patient showed bilateral, abnormal, confluent, relatively symmetric fluid-attenuated inversion recovery (FLAIR)/T2 signal present in the white matter of the cerebral hemispheres; prominent bilateral symmetric frontal convexity involvement; and prominent anterior temporal lobe involvement with relative sparing in the medial temporal lobes. Additional bilateral, confluent and punctate, scattered, fairly symmetric signal foci throughout the frontal parietal white matter tracks were also noted.

There are currently no disease-modifying therapies available for CADASIL, and treatment is mainly directed toward symptoms and prophylaxis. Patients presenting with symptoms of TIA and acute TIA are managed according to the guidelines of stroke medicine; secondary prophylaxis treatment is with statins, antiplatelet therapy, blood pressure reduction, and smoking cessation [12]. Our patient was treated in the inpatient setting for TIA as per the guidelines of stroke medicine and has been discharged on aspirin, statin, and medications for migraine prophylaxis and nonsteroidal anti-inflammatory drugs (NSAIDs) for any acute symptoms of migraine.

We conclude by underlining that CADASIL syndrome should be suspected in individuals who have TIAs or acute strokes and have no risk factors, family history of stroke, or specific neuroimaging findings. Further testing is warranted with *NOTCH3* gene mutation testing.

## Conclusions

CADASIL is one of the important causes of stroke and vascular dementia in the younger population. The diagnosis of CADASIL can be made by personal and family history, skin biopsy showing the accumulation of granular osmiophilic material, and MRI of the head showing high-intensity signal lesions, microbleeds, and white matter changes. CADASIL has no specific treatment; therefore, care focuses on lowering risk factors such as diabetes and hypertension, as well as symptom control. This article aims to highlight rare and inherited causes of TIA and strokes in younger patients.
